# Chronic urticaria after Moderna COVID-19 vaccine boosters: A case series

**DOI:** 10.1016/j.jdcr.2023.11.037

**Published:** 2024-01-23

**Authors:** Chenin Ryan, Kevin Li, Raven Bennett, Matthew J. Davis, Marcus Shaker, Karen Hsu Blatman, Sarah Hughes, Julianne A. Mann

**Affiliations:** a Geisel School of Medicine at Dartmouth, Hanover, New Hampshire; b Department of Dermatology, Dartmouth Hitchcock Medical Center, Lebanon, New Hampshire; c Section of Allergy and Clinical Immunology, Dartmouth Hitchcock Medical Center, Geisel School of Medicine at Dartmouth, Lebanon, New Hampshire; d Departments of Dermatology and Pediatrics, Dartmouth Hitchcock Medical Center, Lebanon, New Hampshire

**Keywords:** chronic urticaria, COVID-19, vaccine reaction

## Introduction

Cutaneous reactions to messenger RNA (mRNA) COVID-19 vaccines have been well-documented during the COVID-19 pandemic, with local injection site erythema and angioedema being the most reported skin findings.^1^ Additionally, chronic urticaria (CU), defined as recurrent urticaria lasting at least 6 weeks, has previously been reported in the literature following mRNA COVID-19 vaccination.^2^, ^3^, ^4^, ^5^ In this case series, we add to the growing literature detailing cutaneous mRNA vaccine reactions by describing 7 patients who developed CU in the weeks after receiving the MRNA-1273 vaccine (Moderna) COVID-19 booster vaccine. Two of these patients subsequently received a BNT162b2b vaccine (Pfizer-BioNTech) bivalent booster without any worsening of their CU.

## Case series

Seven patients developed CU after receiving an mRNA COVID-19 vaccine (Table I). The median age was 42 years (range, 20-64 years) and 4 of the 7 patients were female. All the patients received the mRNA-1273 vaccines, with 6 of the 7 developing CU after their third dose and the remaining patient after their fourth dose. Urticaria had a median onset of 11 days following vaccination (range, 10-42 days). Two patients opted to receive an additional dose in 2022 without worsening of their urticaria, choosing an alternative manufacturer (BNT162b2b). One of these patients also chose to receive a dose of the Comirnaty (Pfizer) vaccine in the fall of 2023, without worsening of their background urticaria. Of the 7 patients, 6 were treated with sedating and/or nonsedating H1 antihistamines and 1 patient declined medication. Three patients experienced symptom resolution after a median of 14 months (range, 11-19 months). However, the remaining 4 patients continue to experience urticaria at the time of this report (median duration 16 months; range, 15-17 months).

**Table I:** Clinical data summarizing case highlights

Case #	Age	Sex	Past medical history	First dose	Second dose	Culprit dose	Onset delay (days)	Subsequent COVID-19 doses and interval (months) between last vaccine doses	CU symptoms	Current treatment(s) for CU	CU duration (months)
1	42	F	Eczema, environmental allergies, cold-induced reactive airway disease	mRNA-1273 vaccine	mRNA-1273 vaccine	mRNA-1273 vaccine #3	11	BNT162b2b vaccine #4, 11 mo Comirnaty (Pfizer) 2023-2024 formula vaccine #5, 12 mo	Urticaria, fullness in throat, lip angioedema, dermatographism	Fexofenadine (180 mg every d)	>17 mo
2	60	M	Seasonal allergic rhinitis	mRNA-1273 vaccine	mRNA-1273 vaccine	mRNA-1273 vaccine #3	10	None	Urticaria, dermatographism	Cetirizine (10 mg every d)	19 mo
3	64	F	None	mRNA-1273 vaccine	mRNA-1273 vaccine	mRNA-1273 vaccine #4	12	None	Urticaria, lip angioedema	Levocetirizine (5 mg 3 times a d), hydroxyzine (25 mg 3 times a d)	11 mo
4	62	M	None	mRNA-1273 vaccine	mRNA-1273 vaccine	mRNA-1273 vaccine #3	42	None	Urticaria, dermatographism	Hydroxyzine (25 mg 3 times a d), fexofenadine (180 mg twice a d)	>16 mo
5	21	F	None	mRNA-1273 vaccine	mRNA-1273 vaccine	mRNA-1273 vaccine #3	10	None	Urticaria, lip angioedema, tightness in chest	Fexofenadine (180 mg every d)	>16 mo
6	20	F	Childhood urticaria	mRNA-1273 vaccine	mRNA-1273 vaccine	mRNA-1273 vaccine #3	10	None	Urticaria, dermatographism	Fexofenadine (180 mg twice a d)	>15 mo
7	38	M	None	mRNA-1273 vaccine	mRNA-1273 vaccine	mRNA-1273 vaccine #3	14	BNT162b2b vaccine #4, 12 mo	Urticaria	None	14 mo

*CU*, Chronic urticaria; *mRNA*, messenger RNA.

## Discussion

In the present case series we describe 7 patients, in accordance with the case reports guidelines,^6^ who developed delayed-onset CU 1 to 6 weeks after receiving an mRNA-1273 COVID-19 vaccine booster. Although 1 patient reported subjective mild throat fullness and lip angioedema, there were no instances of anaphylaxis in the 7 reported cases. Of note, 2 patients in our case series subsequently received a BNT162b2b vaccine COVID-19 booster with no exacerbation of their urticaria or dermatographism. It is unknown whether CU after COVID-19 vaccines develops as a result of the immune response to the vaccine mRNA, a protein that is subsequently generated, a vaccine excipient, another component of the vaccine, or for some other reason. While we describe a potential correlation between COVID-19 vaccines and CU, causation cannot be established without further study. Anvari et al reported 60 cases of urticaria or angioedema in the COVID-19 Vaccine Allergy Case Registry after vaccination.^7^ At the time of their publication, only the initial 2-dose series was Food and Drug Administration-approved. They noted that 77% of urticarial reactions occurred after the first vaccine dose, and 47% of reactions were delayed (defined as greater than 24 hours after vaccination).^7^ Notably, the cases we present occurred following an mRNA-1273 COVID-19 vaccine booster. This aligns with previous literature showing Moderna more often causing urticaria and/or angioedema in a delayed pattern when compared to Pfizer.^7^ In 1 report, 20/29 patients who received the Moderna vaccine and developed urticaria experienced symptoms 24 hours or later following their vaccination, vs 8/31 patients who received the Pfizer vaccine and developed urticaria.^7^ This may be attributed to the greater immunogenicity and higher concentration of mRNA delivered with the Moderna vaccine when compared to the Pfizer vaccine and may explain the lessened immunogenic response in those who subsequently received the Pfizer vaccine.^8^ Therefore, it is possible that the 2 patients who received doses of the Pfizer vaccine without any worsening of their urticaria may have tolerated the Pfizer vaccine better due to its lower immunogenicity. Similar to prior reported experience, most of our patients responded well to oral antihistamines.^7^ Although omalizumab may be an effective treatment for CU following COVID-19 vaccination, none of our patients received this therapy.^4^^,^^9^

Most vaccine reactions in general, even immediate allergic reactions, are not contraindications to receiving additional doses of the causative vaccine.^10^, ^11^, ^12^, ^13^ However, postvaccination delayed-onset CU can lead to future vaccine hesitancy.^13^ In a report by Judd et al, 69% of patients with CU indicated they would not receive future COVID-19 vaccination even if recommended.^13^ Still, in our series subsequent COVID-19 vaccine was well tolerated in those patients who chose to receive it. Shared decision making is essential when discussing additional vaccine doses for patients with post-COVID-19 vaccination CU.^14^^,^^15^

The retrospective observational nature of this report limits conclusions that can be drawn from our experience, as it is possible that some patients may develop CU unrelated to vaccination.

## Conclusion

In summary, we highlight 7 patients who developed CU after receiving the mRNA-1273 COVID-19 vaccine boosters. Given that 2 of these patients were able to tolerate BNT162b2b vaccine boosters and 1 tolerated a subsequent Comirnaty 2023 to 2024 formula booster with no exacerbation of their CU, switching vaccine manufacturers could be 1 approach for physicians when discussing options for future vaccinations with patients who have had adverse reactions to COVID-19 boosters or other vaccines; however, the necessity of such a change is uncertain.

## Conflicts of interest

None disclosed.
